# Assessment of the influence of direct tobacco smoke on infection and active TB management

**DOI:** 10.1371/journal.pone.0182998

**Published:** 2017-08-24

**Authors:** Neus Altet, Irene Latorre, María Ángeles Jiménez-Fuentes, José Maldonado, Israel Molina, Yoel González-Díaz, Celia Milà, Esther García-García, Beatriz Muriel, Raquel Villar-Hernández, Maisem Laabei, Andromeda-Celeste Gómez, Pere Godoy, Maria Luiza de Souza-Galvão, Segismundo Solano, Carlos A. Jiménez-Ruiz, Jose Domínguez, the PII Smoking SEPAR Working Group

**Affiliations:** 1 Unitat de Tuberculosi Vall d’Hebron-Drassanes, Hospital Universitari Vall d’Hebron, Barcelona, Spain; 2 Serveis Clínics, Unitat Clínica de Tractament Directament Observat de la Tuberculosi, Barcelona, Spain; 3 Institut d’Investigació Germans Trias i Pujol, CIBER Enfermedades Respiratorias, Universitat Autònoma de Barcelona, Badalona, Spain; 4 Institut de Biotecnologia i Biomedicina, Bellaterra, Barcelona, Spain; 5 Departament de Salut, Generalitat de Cataluña, CIBER Epidemiología y Salud Pública, IRB-Lleida, Universitat de Lleida, Lleida, Spain; 6 Hospital Universitario Gregorio Marañón, Madrid, Spain; 7 Unidad de Tabaquismo de la Comunidad Autónoma de Madrid, Madrid, Spain; University of Cape Town Lung Institute, SOUTH AFRICA

## Abstract

**Background:**

Smoking is a risk factor for tuberculosis (TB) infection and disease progression. Tobacco smoking increases susceptibility to TB in a variety of ways, one of which is due to a reduction of the IFN-γ response. Consequently, an impaired immune response could affect performance of IFN-γ Release Assays (IGRAs).

**Objective:**

In the present study, we assess the impact of direct tobacco smoking on radiological manifestations, sputum conversion and immune response to *Mycobacterium tuberculosis*, analyzing IFN-γ secretion by IGRAs.

**Methods:**

A total of 525 participants were studied: (i) 175 active pulmonary TB patients and (ii) 350 individuals coming from contact tracing studies, 41 of whom were secondary TB cases. Clinical, radiological and microbiological data were collected. T-SPOT.TB and QFN-G-IT were processed according manufacturer’s instructions.

**Results:**

In smoking patients with active TB, QFN-G-IT (34.4%) and T-SPOT.TB (19.5%) had high frequencies of negative results. In addition, by means of an unconditional logistic regression, smoking was a main factor associated with IGRAs’ false-negative results (aOR: 3.35; 95%CI:1.47–7.61; p<0.05). Smoking patients with active TB presented a high probability of having cavitary lesions (aOR: 1.88; 95%CI:1.02–3.46;p<0.05). Mean culture negativization (months) ± standard deviation (SD) was higher in smokers than in non-smokers (2.47±1.3 *versus* 1.69±1.4). Latent TB infection (LTBI) was favored in smoking contacts, being a risk factor associated with infection (aOR: 11.57; 95%CI:5.97–22.41; p<0.00005). The IFN-γ response was significantly higher in non-smokers than in smokers. Smoking quantity and IFN-γ response analyzed by IGRAs were dose-dependent related.

**Conclusions:**

Smoking had a negative effect on radiological manifestations, delaying time of sputum conversion. Our data establish a link between tobacco smoking and TB due to a weakened IFN-γ response caused by direct tobacco smoke.

## Introduction

Tobacco smoking and tuberculosis (TB) remain as two serious global health threats. The World Health Organization has stated that tobacco smoking results in approximately 6 million deaths annually, and that diseases associated with smoking claim more lives than HIV, malaria and TB together. In addition, TB killed 1.4 million people in 2015 [1, 2]. Currently, the association between tobacco and TB has been underestimated because available studies have not conclusively provided a confirmatory link. However, recent investigations suggest that tobacco smoking has a negative impact on TB outcome, resulting in delay in culture conversion during therapy and frequently requiring treatment extension [3–9].

Several studies indicate that active and passive tobacco smoke exposure are risk factors for latent tuberculosis infection (LTBI) and active TB progression. In addition, smoking has been associated with cavitary lesions, bacillary load, smear conversion delay, and high risk of reactivation and death during or after treatment. Of note, the relative risk of TB reactivation in smokers is comparable with respect to the risk observed in end-stage renal disease patients or individuals under anti-Tumour Necrosis Factor (TNF)-alpha (α) therapy [3–5, 10, 11].

T-cells producing Interferon (IFN)-gamma (γ) have a key role in the protective immunity against *Mycobacterium tuberculosis*. IFN-γ Release Assays (IGRAs), introduced a decade ago, are based on the detection of IFN-γ secreted by sensitized T-cells in peripheral blood after stimulation with specific *M*. *tuberculosis* antigens. The two current assays based on this technology, approved by U.S. Food and Drug Administration (FDA) and European Commission (EC) are QuantiFERON-TB Gold (QFN-G-IT, QIAGEN, Dusseldorf, Germany) and T-SPOT.TB (Oxford Immunotec, Abingdon, UK). Both assays are useful approaches for LTBI diagnosis because the specific antigens used in both technologies avoid cross-reaction with BCG-vaccine and most of non-tuberculous mycobacteria (NTM) [12–15].

The effect of tobacco smoking on the immune system is not completely understood. It has been demonstrated using murine models infected with *M*. *tuberculosis* that tobacco smoke increases susceptibility to TB as a result of diminished recruitment of IFN-γ producing T-cells to the lungs and spleens [16–18]. According to these studies, clinical performance of IGRAs, based on IFN-γ secreting T-cells detection, could be affected by the impaired immune response as a consequence tobacco smoke exposure. Consequently, their use in such kind of population may help clinicians to understand the immune status of the patient and to link it with tobacco smoke.

The present study investigates the immune response against *M*. *tuberculosis* studied by IGRAs, in smoking and non-smoking patients with active TB and LTBI-exposed contacts, as well as the impact of smoking on radiological manifestations and microbiological evolution in TB patients.

## Materials and methods

### Study design and sample collection

This is a prospective and cross-sectional study. Patients were recruited from June 2013 to May 2014. Data on demographic and clinical parameters were obtained through a questionnaire during control routine consultation or/and at the moment of the inclusion. Active TB patients and individuals coming from contact tracing studies were included. Patients were recruited in Serveis Clínics [specialized center on Direct Observed Therapy (DOT) located in Barcelona] and in the Unitat de Tuberculosi Vall d’Hebron-Drassanes (Hospital Universitari Vall d’Hebron, Barcelona). A respiratory sample was obtained in all active pulmonary TB patients for the diagnosis and control of the disease.

A total of 11mL of blood was drawn for performing the two IGRAs in all study participants. Blood was collected at the same time of TST testing. Blood for T-SPOT.TB and QFN-G-IT was directly sent to Institut d’Investigació Germans Trias i Pujol for assay testing. This study has been approved by the Ethical Committee of the Institut d’Investigació en Atenció Primària (IDIAP) Jordi Gol in Barcelona. Informed consent was obtained for patient participation. All the data regarding patient identification and information was handled in a confidential manner and in accordance with the Spanish Law 15/1999 on the Protection of Personal Character Data.

### Study population

The participants in this study included active pulmonary TB patients scheduled for anti-TB therapy initiation and individuals coming from contact tracing studies. Patient groups were defined based on the following criteria: (i) active pulmonary TB patients with microbiologic confirmation by culture, a compatible radiography with the disease and good clinical response to anti-TB chemotherapy. (ii) Asymptomatic individuals coming from contact tracing studies where the index case was smear and culture positives. LTBI was defined in this population as having positive IGRAs (T-SPOT.TB and/or QFN-G-IT) and a chest radiography without alterations. Exclusion criteria were having a previous known contact, a prior documented positive TST and an anti-TB therapy prescription in the past. All patients included in the present study were tested with both IGRAs (QFN-G-IT and T-SPOT.TB).

### Technical procedures

TST was performed according the Mantoux technique using two tuberculin units of PPD RT23 (Statens Serum Institut, Copenhagen, Denmark), and was evaluated within 48-72h by trained nurses and doctors. All TST≥5mm were classified as a positive result independently of the BCG status according to the Spanish Pneumology and Thoracic Surgery Society guidelines [19]. T-SPOT.TB and QFN-G-IT were processed and interpreted according manufacturer’s instructions provided in the kits.

### Study variables

Clinical (symptoms and pathology), radiological and microbiological (smear and culture) data was recorded. Presence of comorbidities was analyzed for diabetes, HIV co-infection and chronic obstructive pulmonary disease (COPD). Other pathologies as end-stage renal disease, pancreatitis, psychological disorders and hepatitis were grouped together as “comorbidities”. Tobacco consumption was investigated by means of two independent interviews. Smoking quantity was classified as a standard “pack-years ratio” defined as: (number of cigarettes consumed per day/20) × (number of years the person has smoked) [20]. “Underweight” was considered when the variable Body Mass Index (BMI) was ≤18.5 [21]. The Social Class variable was categorized according to the Spanish Occupational National Center (CNO) [22].

### Statistical analysis

Qualitative variables are based on the calculation of the number and its percentage. Quantitative variables are based on the calculation of the median and the Interquartile Range (IQR). The chi-square test and Fisher’s exact test have been used to compare qualitative variables. The Odds Ratios (OR) and its 95% confidence intervals (CI) were calculated for the associated risk between variables; the associated variables with a value p<0.05 were analyzed at a multivariate level by means of a logistic regression and an estimation of an Adjusted Odds Ratio (aOR). Non-parametric tests (Mann-Whitney, Kolmogorov-Smirnov, Kruskal-Wallis) have been used to compare quantitative variables according to the categories of the group variable. Sensitivity, specificity, likelihood ratios (LR), positive and negative predictive values (PPV and NPV), pre-test and post-test probability of developing active TB were calculated. Data was analyzed with Epi Info 7.1.2 (www.cdc.gov/epiinfo/7). The overall RD1 response in T-SPOT.TB was calculated as the sum of the spot forming cells (SFCs) obtained in ESAT-6 and CFP-10 panels.

## Results

### Patient characteristics

A total of 525 participants were studied: (i) 175 active pulmonary TB patients and (ii) 350 individuals coming from contact tracing studies, 41 of whom were secondary TB cases (a total of 309 contacts without active TB). Globally, 62.1% (326/525) were men. Mean age (years) ± standard deviation (SD) was 34.00 ± 13.2. The proportion of tobacco smokers was significantly higher in TB patients (59.3%; 128/216) with respect to contacts (43%; 133/309; p<0.001). Daily alcohol consumption frequency was not significant between groups; however, consumption of >40g/day was higher in active TB patients (25.9%; 56/216) than in contacts (0.6%; 2/309). Moreover, frequency of social class IV-VI, intravenous drug use (IVDU) and comorbidities such as diabetes, COPD or HIV co-infection were also higher in TB diseased patients *versus* contacts (Tables 1 and 2).

**Table 1:** Main demographic characteristics and risk factors according to TST and IGRAs results in TB diseased patients.

Variables	N (%)	TST ≥5mm		QFN-G-IT Positive		T-SPOT.TB Positive	
		N (%)	p-value	N (%)	p-value	N (%)	p-value
**Total**	216 (100)	197 (91.2)		158 (73.1)		184 (85.2)	
**Sex**							
Male	163 (75.5)	149 (91.4)	NS	120 (73.6)	NS	143 (87.7)	NS
Female	53 (24.5)	48 (90.6)		38 (71.1)		41 (77.4)	
**BCG**							
Yes	158 (73.1)	149 (94.3)	<0.05	125 (79.1)	<0.01	139 (88.0)	NS
No	58 (26.9)	48 (82.8)		33 (56.9)		45 (77.6)	
**SC IV-VI**							
Yes	174 (80.6)	159 (91.4)	NS	130 (74.7)	NS	146 (83.9)	NS
No	42 (19.4)	38 (90.5)		28 (66.7)		38 (90.5)	
**Employed**							
Yes	172 (79.6)	159 (92.4)	NS	134 (77.9)	<0.005	149 (86.6)	NS
No	44 (20.4)	38 (86.4)		24 (54.5)		35 (79.5)	
**Diagnostic delay**							
<50 days	129 (59.7)	116 (89.9)	NS	99 (76.7)	NS	111 (86.0)	NS
≥50 days	87 (40.3)	81 (93.1)		59 (67.8)		73 (83.9)	
**Immigrant**							
Yes	145 (67.1)	140 (96.6)	<0.001	120 (82.8)	<0.001	133 (91.7)	<0.01
No	71 (32.9)	57 (80.3)		38 (53.5)		51 (71.8)	
**Underweight**							
Yes	37 (17.2)	32 (86.5)	NS	23 (62.2)	NS	28 (75.7)	NS
No	178 (82.8)	164 (92.1)		134 (75.3)		155 (87.1)	
**Alcohol >40g/day**							
Yes	56 (25.9)	46 (82.1)	<0.01	34 (60.7)	<0.05	41 (73.2)	<0.01
No	160 (74.1)	151 (94.4)		124 (77.5)		143 (89.4)	
**IVDU**							
Yes	22 (10.2)	14 (63.6)	<0.005	9 (40.9)	<0.001	13 (59.1)	<0.005
No	194 (89.8)	183 (94.3)		149 (76.8)		171 (88.1)	
**Smoking**							
Yes	128 (59.3)	115 (89.8)	NS	84 (65.6)	<0.001	103 (80.5)	<0.05
No	88 (40.7)	82 (93.2)		74 (84.1)		81 (92.0)	
**Other co-morbidities**							
Yes	90 (41.7)	72 (80.0)	<0.0001	51 (56.7)	<0.001	64 (71.1)	<0.001
No	126 (58.3)	125 (99.2)		107 (84.9)		120 (95.2)	
**Diabetes**							
Yes	12 (5.6)	11 (91.7)	NS	9 (75.0)	NS	11 (91.7)	NS
No	204 (94.4)	186 (91.2)		149 (73.0)		173 (84.8)	
**COPD**							
Yes	27 (12.5)	24 (88.9)	NS	11 (40.7)	<0.001	18 (66.7)	<0.05
No	189 (87.5)	173 (91.5)		147 (77.8)		166 (87.8)	
**HIV**							
Yes	13 (6.0)	2 (15.4)	<0.0001	2 (15.4)	<0.001	5 (38.5)	<0.001
No	203 (94.0)	195 (96.1)		156 (76.8)		179 (88.2)	

TB: tuberculosis; NS: non-significant; TST: tuberculin skin test; SC: social class; IVDU: intravenous drug users; COPD: chronic obstructive pulmonary disease; HIV: human immunodeficiency virus

**Table 2:** Main demographic characteristics and risk factors associated with TST and IGRAs positivity in individuals coming from contact tracing studies (including secondary TB cases).

Variables	N (%)	TST ≥5mm		QFN-G-IT Positive		T-SPOT.TB Positive	
		N (%)	p-value	N (%)	p-value	N (%)	p-value
**Total**	350 (100)	245 (70.0)		131 (37.4)		155 (44.3)	
**Sex**							
Male	190 (54.3)	137 (72.1)	NS	78 (41.1)	NS	92 (48.4)	NS
Female	160 (45.7)	108 (67.5)		53 (33.1)		63 (39.4)	
**BCG**							
Yes	258 (73.7)	195 (75.6)	<0.0005	101 (39.1)	NS	114 (44.2)	NS
No	92 (26.3)	50 (54.3)		30 (32.6)		41 (44.6)	
**SC IV-VI**							
Yes	160 (45.7)	117 (73.1)	NS	74 (46.3)	<0.005	88 (55.0)	<0.0005
No	190 (54.3)	128 (67.4)		57 (30.0)		67 (35.3)	
**Immigrant**							
Yes	220 (62.9)	160 (72.7)	NS	89 (40.5)	NS	103 (46.8)	NS
No	130 (37.1)	85 (65.4)		42 (32.3)		52 (40.0)	
**Alcohol Daily**^a^							
Yes	146 (41.8)	112 (76.7)	<0.05	74 (50.7)	<0.00005	85 (58.2)	<0.00005
No	203 (58.2)	133 (65.5)		57 (27.9)		70 (34.3)	
**IDVU**							
Yes	3 (0.9)	3 (100.0)	NS	1 (33.3)	NS	2 (66.7)	NS
No	349 (99.1)	242 (69.9)		131 (37.7)		154 (44.4)	
**Smoking**							
Yes	162 (46.3)	138 (85.2)	<0.0001	97 (59.9)	<0.0001	116 (71.6)	<0.0001
No	188 (53.7)	107 (56.9)		34 (18.1)		39 (20.7)	
**Other Comorbidities**							
Yes	34 (9.7)	31 (91.2)	<0.01	19 (55.9)	<0.05	21 (61.8)	<0.05
No	316 (90.3)	214 (67.7)		112 (35.4)		134 (42.4)	
**Diabetes**							
Yes	0 (0.0)	0 (0.0)	—	0 (0.0)	—	0 (0.0)	—
No	350 (100.0)	245 (70.0)		131 (37.4)		155 (44.3)	
**COPD**							
Yes	3 (0.9)	3 (100.0)	NS	2 (66.7)	NS	3 (100.0)	NS
No	347 (99.1)	242 (69.9)		129 (37.2)		152 (40.9)	
**HIV**							
Yes	3 (0.9)	3 (100.0)	NS	2 (66.7)	NS	1 (33.3)	NS
No	347 (99.1)	242 (69.7)		129 (37.2)		154 (44.4)	
**IC Smoker**							
Yes	228 (65.1)	167 (73.2)	NS	101 (44.3)	<0.0005	119 (52.2)	<0.0001
No	122 (34.9)	78 (63.9)		30 (24.6)		36 (29.5)	
**Living together with IC**							
Yes	126 (36.0)	89 (70.6)	NS	64 (50.8)	<0.0005	77 (61.1)	<0.00005
No	224 (64.0)	156 (69.6)		67 (29.9)		78 (34.8)	
**Exposed <50 days**							
Yes	303 (86.6)	207 (68.3)	NS	103 (34.0)	<0.005	121 (39.9)	<0.0001
No	47 (13.4)	38 (80.9)		28 (59.6)		34 (72.3)	

^a^ Two of the contacts (0.6%) consumed >40g/day of alcohol

IC: index case; NS: non-significant; TB: tuberculosis; TST: tuberculin skin test; SC: social class; IVDU: intravenous drug users; COPD: chronic obstructive pulmonary disease; HIV: human immunodeficiency virus

### IGRAs performance on active TB patients

Table 1 indicates the main demographic and risk factors according to TST and IGRAs results in active TB patients. Frequency of positive QFN-G-IT results were lower than that obtained for T-SPOT.TB (73.1% [158/216] by QFN-G-IT *versus* 85.2% [184/216] by T-SPOT.TB; p<0.005). Globally, active TB patients presented seven indeterminate results by any of both IGRAs; five of them corresponded to smokers. A total 119 active TB patients lost weight as a diseased symptom. In addition, the 17.2% (37/216) of these diseased patients presented a BMI ≤18.5. However, BMI variable had no significant differences on IGRAs’ positivity.

Because of the sensitivity of IGRAs in active TB patients is not 100%, the frequency of false-negative results in this group was analyzed based on tobacco smoking. T-SPOT.TB and QFN-G-IT presented 19.5% (25/128) and 34.4% (44/128) of false-negative results respectively among the smoker group, while only 7.95% (7/88) and 15.9% (14/88) of false-negative results were observed in non-smokers, respectively. By means of an unconditional logistic regression, the main factors associated with a false-negative results were: smoking (aOR: 3.35; 95%CI:1.47–7.61; p<0.05); comorbidities (aOR:32.55; 95%CI:1.18–5.52; p<0.05), diagnostic delay < 50 days (aOR: 0.40; 95%C.I:0.20–0.79; p<0.005) and immunosuppression (aOR:13.36; 95%CI:1.34–132.52; p<0.05).

### Radiological manifestations and microbiological evolution of active TB patients

Table 3 shows the associations between different TB risk factors and clinical characteristics of the disease. Smoking patients with active TB presented a high probability of having cavitary lesions (aOR: 1.88; 95%C.I:1.02–3.46; p<0.005). There were not significant differences between smokers and non-smokers with respect to the sputum smear result (variable “smear-positive”). The mean culture negativization (months) ± SD was significantly later in smokers than in non-smokers (2.47±1.3 *versus* 1.69±1.4; excluding 16 cases with drug-resistances); furthermore, days of culture negativization significantly increased when cigarette dose augmented (p<0.001). Based on this fact the need of extending anti-TB chemotherapy in smokers over non-smokers was required (OR: 3.1; 95%CI:1.13–8.48; p<0.05). In addition, alcohol consumption >40g/day was observed as a risk factor for being smear-positive (aOR: 4.96; 95%CI:1.99–12.33); p<0.001) and having bilateral lesions (aOR for unilateral lesions: 0.37; 95%CI:0.16–0.83; p<0.05). Underweight was a risk factor significantly associated with culture positivity delay (aOR for culture negativization: 0.47; 95%CI:0.19–1.10; p<0.05) and bilateral lesions (aOR for unilateral lesions: 0.41; 95%CI:0.18–0.94; p<0.05).

**Table 3:** Associations between different TB risk factors and clinical characteristics of the disease. A multivariate analysis and an aOR has been performed considering statistically significant variables observed in the bivariate analysis.

Variables	Cavitary			Smear-positive			Unilateral			Culture negativization (2nd month)		
	N (%)	aOR (95% CI)	p-value	N (%)	aOR (95% CI)	p-value	N (%)	aOR (95% CI)	p-value	N (%)	aOR (95% CI)	p-value
**Sex**												
Female	19 (35.8)	1	NS	32 (60.4)	NA	--	39 (73.6)	NA	--	41 (77.3)	NA	--
Male	85 (52.2)	1.39 (0.80–3.20)		107 (65.6)			111 (68.1)			116 (71.2)		
**Diagnostic delay**												
≥50 days	49 (56.3)	1	NS	67 (77.0)	1	<0.005	57 (65.5)	NA	--	57 (65.5)	1	NS
<50 days	54 (42.5)	0.60 (0.34–1.06)		71 (55.9)	0.35 (0.19–0.66)		92 (72.4)			99 (79.8)	1.90(0.95–3.78)	
**Alcohol >40g/day**												
No	69 (43.1)	1	NS	90 (56.3)	1	<0.001	125 (78.1)	1	<0.05	124 (79.0)	1	NS
Yes	35 (62.5)	1.58 (0.78–3.22)		49 (87.5)	4.96 (1.99–12.33)		25 (44.6)	0.37 (0.16–0.83)		33 (58.9)	0.64 (0.3–1.36)	
**Smoking**												
No	31 (35.2)	1	<0.05	50 (56.8)	NA	--	71 (80.7)	1	NS	76 (86.4)	1	<0.05
Yes	73 (57.0)	1.88 (1.02–3.46)		89 (69.5)			79 (61.7)	0.63 (0.31–1.30)		81 (64.8)	0.36 (0.15–0.82)	
**IVDU**												
No	91 (46.9)	NA	--	121 (62.4)	NA	--	139 (71.6)	1	NS	144 (75.4)	NA	--
Yes	13 (59.1)			18 (81.8)			11 (50.0)	1.27 (0.43–3.74)		13 (59.1)		
**BCG**												
No	30 (51.7)	NA	--	43 (74.1)	1	NS	33 (56.9)	1	NS	40 (69.0)	NA	--
Yes	74 (46.8)			96 (60.8)	1.10 (0.49–2.48)		117 (74.1)	0.78 (0.24–2.50)		117 (75.5)		
**Immigrant**												
No	37 (52.1)	NA	--	51 (71.8)	NA	--	38 (53.5)	1	NS	48 (67.6)	NA	--
Yes	67 (46.2)			88 (60.7)			112 (77.2)	1.83 (0.55–6.13)		109 (76.8)		
**Social Class IV-VI**												
No	21 (50.0)	NA	--	26 (61.9)	NA	--	30 (71.4)	NA	--	30 (75.0)	NA	--
Yes	82 (47.4)			112 (64.7)			120 (69.4)			127 (73.8)		
**HIV positive**												
No	101 (49.8)	NA	--	131 (64.5)	NA	--	144 (70.9)	NA	--	144 (72.0)	NA	--
Yes	3 (23.1)			8 (61.5)			6 (46.2)			13 (100)		
**Diabetes**												
No	98 (48.0)	NA	--	128 (62.7)	1	NS	142 (69.6)	NA	--	152 (75.6)	1	<0.01
Yes	6 (50.0)			11 (91.7)	7.7(0.93–63.8)		8 (66.7)			5 (41.7)	0.16 (0.04–0.61)	
**Other Comorbidities**												
No	59 (46.8)	NA	--	75 (59.5)	NA	--	98 (77.8)	1	NS	96 (77.4)		
Yes	45 (50.0)			64 (71.1)			52 (57.8)	0.79 (0.35–1.75)		61 (68.5)	NA	
**COPD**												
No	88 (46.6)	NA	--	116 (61.4)	1	NS	137 (72.5)	1	NS	141 (75.8)	NA	--
Yes	16 (59.3)			23 (85.2)	1.70 (0.50–5.81)		13 (48.1)	1.28 (0.44–3.71)		16 (59.3)		
**Underweight**												
No	80 (44.9)	NA	--	110 (61.8)	NA	--	132 (74.2)	1	<0.05	137 (78.3)	1	<0.05
Yes	23 (62.2)			28 (75.7)			17 (45.9)	0.41 (0.18–0.94)		20 (54.1)	0.47 (0.19–1.10)	

NA: non-applicable. Non-significant variables in the bivariate analysis were non-applicable in the multivariate analysis; TB: tuberculosis; aOR: adjusted Odds Ratio; CI: confidence interval; NS: non-significant; COPD: chronic obstructive pulmonary disease; IVDU: intravenous drug users; HIV: human immunodeficiency virus; COPD: chronic obstructive pulmonary disease

### Likelihood ratios and predictive values for active TB diagnosis

Both LRs and PVs were calculated in order to estimate the active TB diagnostic accuracy of TST, QFN-G-IT and T-SPOT.TB. As shown in Table 4, positive LRs observed in smoking patients were 1.09, 1.16 and 1.18 by TST, QFN-G-IT and T-SPOT.TB respectively. In contrast, positives LR values obtained in non-smoking patients were 1.73, 6.43 and 6 by TST, QFN-G-IT and T-SPOT.TB respectively. Similarly, PPVs for IGRAs were higher in non-smokers than in smokers (QFN-G-IT: PPV in smokers 35.2% *versus* PPV in non-smokers 98.1%; T-SPOT.TB: PPV in smokers 50.8% *versus* PPV in non-smokers 98.9%). In addition, negative LRs and NPV for IGRAs were higher in smokers than in non-smokers. Negative LRs for QFN-G-IT and T-SPOT.TB in smokers were 4.38 and 6.88 times as high as in non-smokers (QFN-G-IT: negative LR in smokers 0.79 *versus* negative LR in non-smokers 0.18; T-SPOT.TB: negative LR in smokers 0.62 *versus* negative LR in non-smokers 0.09).

**Table 4:** Likelihood ratios, pre- and post-Test probabilities of TST, QFN-G-IT and T-SPOT.TB in smoker and non-smoker patients.

Test	TB cases	Non TB cases	Sensitivity (95% CI)	Specificity (95% CI)	PPV % (95% CI)	NPV % (95% CI)	LR positive (95% CI)	LR negative (95% CI)	Positive Pre-Test Probability	Positive Post-Test Probability	Negative Post-Test Probability
**Smoker**											
TST ≥ 5mm	115	110	0.9	0.17	49.01	94.31	1.09	0.59	0.49	0.51	0.36
TST < 5mm	13	23	(0.85–0.95)	(0.11–0.24)	(0.0–86.42)	(86.4–100.0)	(0.99–1.2)	(0.31–1.11)			
**Non-smoker**											
TST ≥ 5mm	82	95	0.93	0.46	92.18	91.16	1.73	0.15	0.33	0.46	0.07
TST < 5mm	6	81	(0.88–0.98)	(0.39–0.53)	(76.8–99.4)	(86.6–94.8)	(1.49–2)	(0.07–0.33)			
**Smoker**											
QFN.G-IT pos	84	75	0.66	0.44	35.24	95.62	1.16	0.79	0.49	0.53	0.43
QFN.G-IT neg	44	58	(0.57–0.74)	(0.35–0.52)	(0.00–74.5)	(86.7–100.0)	(0.96–1.41)	(0.58–1.07)			
**Non-smoker**											
QFN.G-IT pos	74	23	0.84	0.87	98.1	64.9	6.43	0.18	0.33	0.74	0.36
QFN.G-IT neg	14	153	(0.76–0.92)	(0.82–0.92)	(94.0–99.8)	(32.1–79.2)	(4.35–9.52)	(0.11–0.3)			
**Smoker**											
T-SPOT.TB pos	103	91	0.80	0.32	50.77	93.62	1.18	0.62	0.49	0.53	1.5
T-SPOT.TB neg	25	42	(0.74–0.87)	(0.24–0.39)	(0.00–82.7)	(85.5–100.0)	(1.02–1.16)	(0.4–0.95)			
**Non-smoker**											
T-SPOT.TB pos	81	27	0.92	0.85	98.95	67.02	6.0	0.09	0.33	0.75	0.05
T-SPOT.TB neg	7	149	(0.86–0.98)	(0.79–0.9)	(96.3–99.9)	(40.2–79.5)	(4.22–8.54)	(0.05–0.19)			

Pos: positive; neg: negative; TB: tuberculosis; PPV: positive predictive value; NPV: negative predictive value; LR: likelihood ratio; CI: confidence interval

### IGRAs performance in TB contact individuals

**Fig 1:**
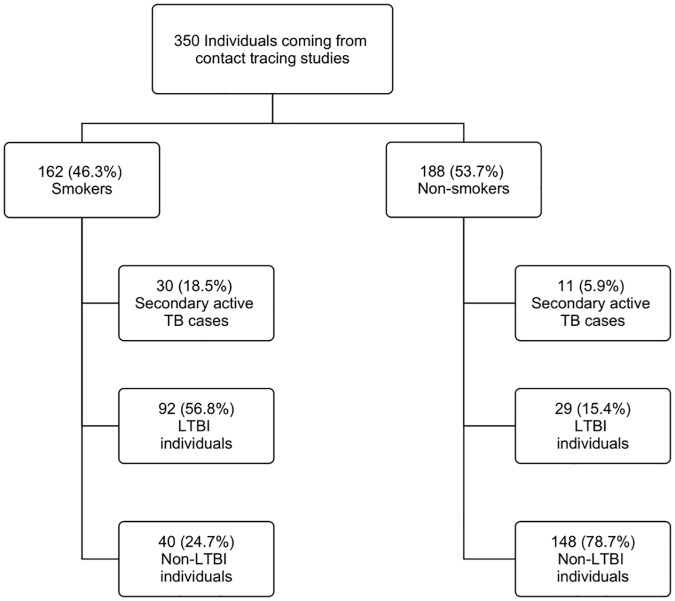
Final diagnosis of all the individuals recruited during contact tracing studies. LTBI, non-LTBI and secondary active TB cases were stratified regarding their smoking or non-smoking condition. LTBI was defined as having positive IGRAs (T-SPOT.TB and/or QFN-G-IT) and a chest radiography without alterations. Active TB cases presented microbiologic confirmation by culture, a compatible radiography with the disease and good clinical response to anti-TB chemotherapy.

Table 2 indicates the possible risk factors associated with TST and IGRAs positivity in individuals coming from contact tracing studies (including the secondary TB cases). A total of 350 individuals were recruited during contact tracing studies, of whom LTBI was diagnosed in a 39.2% (121/309) by QFN-G-IT and/or T-SPOT.TB. In addition, secondary active TB were found in 11.7% (41/350) of the cases. No indeterminate results were obtained in the group of contacts. As expected, positive results obtained by TST were significantly higher in BCG individuals coming from contact tracing studies with respect to non-BCG contacts (p<0.0005), indicating that the vaccine influences TST result. There were no significant differences on the age (years ± SD) between LTBI and non-LTBI individuals (31.7±12.5 *versus* 30.3±11.3). The group of contacts presented 10.3% (36/350) of discordant QFN-G-IT/T-SPOT.TB results (30 cases were QFN-G-IT negative/T-SPOT.TB positive, and six cases were QFN-G-IT positive/T-SPOT.TB negative). By means of an unconditional logistic regression, being a smoker was the unique variable (from all variables analyzed in Table 2) related with IGRAs’ discordant results (aOR: 5.42; 95% CI:1.86–15.79; p<0.005). Furthermore, smoking contacts presented a significantly higher LTBI prevalence when compared with non-smokers (p<0.0001). Afterwards, LTBI risk factors were studied by bivariate and multivariate analysis (Table 5). Smoking (aOR: 11.24; 95%CI:5.78–21.85; p<0.00005), having a daily contact of >6h (aOR: 1.90; 95%CI:1.03–3.49; p<0.05) and being a contact of a smoking index case (aOR: 1.92; 95%CI:1.04–3.55; p<0.05) were the main risk factors associated with LTBI. Moreover, LTBI, non-LTBI and secondary active TB cases found during contact tracing studies were stratified regarding smoking or non-smoking condition. Interestingly, the percentage of secondary active TB cases and LTBI individuals was higher in smokers *versus* non-smokers (secondary active TB: 18.5% in smokers *vs*. 5.9% in non-smokers; LTBI: 56.8% in smokers *vs*. 15.4% in non-smokers; Fig 1).

**Table 5:** LTBI risk factors analysed by bivariate and multivariate analysis.

Variables	LTBI^a^	Bivariate Analysis		Multivariate Analysis	
	N (%)	aOR (95% CI)	p-value	aOR (95% CI)	p-value
**Total**	121 (39.2)				
**Sex**					
Female	54 (37.0)	1	NS	--	--
Male	67 (41.1)	1.19 (0.75–1.88)			
**BCG**					
No	33 (39.8)	1	NS	--	--
Yes	88 (38.9)	0.96 (0.57–1.63)			
**SC IV-VI**					
No	59 (33.1)	1	<0.01	1	NS
Yes	62 (47.3)	1.81 (1.14–2.88)		1.51 (0.84–2.70)	
**Immigrant**					
No	43 (36.4)	1	NS	--	--
Yes	78 (40.8)	1.20 (0.75–1.93)			
**Alcohol Daily**					
No	49 (27.2)	1	<0.00005	1	NS
Yes	72 (56.3)	3.44 (2.13–5.55)		1.00 (0.51–1.94)	
**Smoking**					
No	29 (16.5)	1	<0.00005	1	<0.00005
Yes	92 (69.3)	11.74 (6.59–21.05)		11.57 (5.97–22.41)	
**Other co-morbidities**					
No	109 (37.8)	1	NS	--	--
Yes	12 (57.1)	2.19 (0.89–5.36)			
**HIV (+)**					
No	119 (38.9)	1	NS	--	--
Yes	2 (66.7)	3.14 (0.28–35.04)			
**IC Smoker**					
No	27 (24.1)	1	<0.00005	1	<0.05
Yes	94 (47.7)	2.87 (1.72–4.81)		1.94 (1.05–3.57)	
**Daily >6 hours**					
No	64 (34.4)	1	<0.05	1	<0.05
Yes	57 (46.3)	1.64 (1.03–2.62)		1.81 (1.01–3.31)	
**Exposed <50 days**					
No	20 (64.5)	1	<0.005	1	NS
Yes	101 (36.3)	0.31 (0.14–0.68)		0.56 (0.21–1.50)	

^a^ LTBI was defined in this population as having positive IGRAs (T-SPOT.TB and/or QFN-G-IT) and a chest radiography without alterations.

LTBI: latent tuberculosis infection; aOR: adjusted Odds Ratio; CI: confidence interval; SC: social class; IC: index case; NS: non-significant.

### Impact of tobacco smoking on *M*. *tuberculosis* IFN-γ immune response

The impact of smoking on *M*. *tuberculosis* immune response was analyzed as the amount of IFN-γ secreted (UI/mL) in QFN-G-IT and as the number of T-cells producing IFN-γ (SFCs) in T-SPOT.TB (Table 6). In active TB patients, the response for both assays was significantly higher in non-smokers than in smokers. In LTBI individuals there were numerically more positives in the smoker group based on the diagnosis with IGRAs. However, IFN-γ responses were higher in non-smokers than in smokers. IFN-γ response was related with the smoking dose and it was quantified as pack-years. As a result, a dose-dependent relation was observed between the smoking quantity and the IFN-γ response analyzed by QFN-G-IT and T-SPOT.TB for active TB patients and LTBI contacts. This immune response significantly decreased when the pack-years consumption augmented, with the exception of T-SPOT.TB, where the difference in LTBI individuals is not significant (Table 6).

**Table 6:** Impact of tobacco smoking on *Mycobacterium tuberculosis* immune response.

	N (%)	QFN-G-IT (IU/ml)		T-SPOT.TB (SFC)	
		Median (IQR)	p-Value	Median (IQR)	p-Value
**Active TB**					
**Total**	216 (100)	2.15 (0.30–6.27)		58.50 (16.00–111.00)	
**Smoking**					
*Yes*	128 (59.3)	0.93 (0.20–3.35)	<0.0001	38.5 (13.00–99.00)	<0.0005
*No*	88 (40.7)	3.37 (1.44–9.54)		75.5 (30.00–134.00)	
**Pack-years**					
*None*	88 (40.7)	3.37 (1.44–9.54)	<0.0005	75.5 (30.00–134,00)	<0.01
*1–5*	16 (7.4)	2.54 (0.64–5.65)		59.0 (12.00–101.00)	
*6–15*	21 (9.7)	1.71 (0.25–3.22)		47.00 (16.50–105.00)	
*>15*	91 (42.1)	0.87 (0.13–3.02)		38.50 (13.00–83.00)	
**LTBI contacts**					
**Total**	121 (100)	1.61 (0.65–5.34)		33.00 (18.00–66.00)	
**Smoking**					
*Yes*	93 (76.9)	1.29 (0.48–4.03)	<0.05	31.00 (17.00–53.00)	<0.05
*No*	28 (23.1)	4.65 (0.78–10.58)		50.00 (25.00–144.00)	
**Pack-years**					
None	28 (23.1)	4.65 (0.78–10.58)	<0.05	50.00 (25.00–144.00)	NS
*1–5*	41 (33.9)	2.10 (0.65–4.78)		38.00 (20.00–57.00)	
*6–15*	35 (28.9)	1.24 (0.35–4.02)		24.00 (15.00–46.00)	
*>15*	17 (14.1)	0.94 (0.83–1.16)		24.00 (15.00–50.00)	

SFC: spot-forming cells; IQR: interquartile range; TB: tuberculosis; LTBI: latent tuberculosis infection.

## Discussion

Reducing mortality caused by tobacco-related diseases such as TB continues to be an important goal in clinical microbiology and public health. We have assessed in the present study how tobacco smoke can influence TB radiological manifestations, sputum culture conversion and the immune response against *M*. *tuberculosis* by means of QFN-G-IT and T-SPOT.TB results. The impact of tobacco smoke was examined in active TB patients and individuals coming from contact tracing studies. Our results demonstrate that smoking was associated with cavitary and bilateral radiological findings, culture positivity delay and IGRAs false-negative results in active TB patients. Furthermore, smoking was a risk factor for LTBI in contact individuals. Interestingly, a decreased IFN-γ response was observed in smokers. This response was dose-dependent, with increasing pack-years associated with decreased IFN-γ response.

IGRAs’ sensitivity is limited during active TB [23–25]; however, the presence of false-negative results is even higher in patients who smoke most likely due to the altered inflammatory response associated with smoking. These findings have been previously shown by Aabye and colleagues, concluding that tobacco smokers with active TB present an impaired QFN-G-IT performance associated with false-negative and indeterminate results [26]. Furthermore, LRs and PVs observed in our study in such population also reinforces these findings. LRs and PVs indicate that tobacco smoke influences IGRAs’ performance and it is associated with false-negative results in smokers [27]. Furthermore, others have also described smoking as an independent factor involved in the risk of LTBI development [28–30]. Therefore, our results on smoking patients with active TB and individuals coming from contact tracing studies firmly support previous findings on QFN-G-IT and illustrate novel ones based on the T-SPOT.TB assay.

It has been experimentally demonstrated that tobacco smoke inhibits the proliferation of IFN-γ producing T-cells coming from the lungs of *M*. *tuberculosis* infected mice [16]. In line with this, studies indicate that alveolar compartments from active TB patients are enriched with a specific T-cell subset (called regulatory T-cells or Tregs) which down-regulates the effector immune response. Moreover, these Treg cells reduce the capacity of alveolar and/or monocyte-derived macrophages to control *M*. *tuberculosis* growth [31]. Interestingly, this regulatory profile is enhanced in smokers’ macrophages producing less effector cytokines than non-smokers after infection with *M*. *tuberculosis* [32, 33]. Altogether, these findings suggest that T-cell functions are highly reduced in smokers and that host defense mechanisms in the lung of individuals exposed to tobacco smoke weakly fight against *M*. *tuberculosis* infection. Our work compliments these observations, specifically that smoking alters the immune response decreasing the number of IFN-γ secreting T-cells in T-SPOT.TB and the amount of this cytokine in QFN-G-IT. Subsequently, smokers have high susceptibility of being LTBI infected if they are exposed to the bacilli. Furthermore, we cannot also discard the possibility of being underestimating LTBI diagnosis in smoking contacts due to immune dysfunction. All contacts with negative and positive IGRAs (who received chemoprophylaxis) within the study were followed-up and none of them developed active TB. This data reflects that IGRAs have a high negative predicting value, which is in accordance with previous findings of our research group [34]. In this study, being a contact of a smoking index case was another risk factor associated with LTBI. This could be attributable to more frequent cough in smoking index TB patients, and as a consequence, a higher transmission of the disease through their close contacts [35].

It is shown here that a negative dose-response relation exits between the amount of cigarettes smoked and the IFN-γ response in active TB patients and LTBI individuals. Therefore, an increment of tobacco smoking produces a reduction of these responses observed by both IGRAs. This inverse correlation was also observed in a previous study using QFN-G-IT in HIV-infected individuals for LTBI detection [36]. The alteration on IFN-γ producing T-cells and/or the secreted levels of this cytokine persists as long as the individual continues smoking. As a consequence, several factors such as re-infections, immune risk adjuvants and self-*M*. *tuberculosis* strain pathogenicity could contribute in triggering TB disease [37]. Indeed, 71.1% (91/128) within our cohort of smokers with active TB, smoked more than 15 pack-years *versus* 18.3% (17/93) of LTBI smokers and 5% (2/40) of non-LTBI smokers. Additionally, pack-years estimation not only depends on cigarette numbers, but also on years the individual has been smoking. Therefore, number of smoking years exacerbates the negative effect of tobacco smoke. In our study, mean age (years) ± SD of active TB patients who smoked >15 pack-years versus those who smoked <15 pack-years was 45.02 ± 11.15 and 33.83±13.7, respectively. The same tendency was observed in LTBI individuals, mean ages (years) ± SD in those who smoked >15 pack-years or <15 pack-years were 40.00±8.1 and 30.00±12.55, respectively.

The impact of smoke cessation on immunity against *M*. *tuberculosis* is controversial. It has been shown in a murine model that tobacco smoke cessation is beneficial to pulmonary TB control and allows a quick recovery of anti-mycobacterial immunity [18]. In contrast, O’Leary et al. observed that an impaired immune response to *M*. *tuberculosis* is maintained in alveolar macrophages from ex-smokers [32]. As found in this study and others [10, 38, 39], smoking affects clinical TB manifestations by increasing cavitary and bilateral radiological findings. The impaired immune response observed in smokers due to decreased IFN-γ cytokine secretion may result in increased susceptibility to sever forms of the disease. In addition, a delay of culture negativization is observed in smokers, this finding is also dose-dependent related and results in prolonged treatment, with increased costs associated with therapy and surveillance. [6, 9, 40, 41].

Besides the important findings obtained here, some limitations need to be addressed. First, a single IFN-γ blood quantification may not be sufficient to characterize the alteration of the immune response as a consequence of direct tobacco smoking. Therefore, it may be encouraging to detect and monitor several cytokines and cell populations in blood and bronchoalveolar lavage of individuals with an impaired immune system such as smokers. Second, the number of patients who stopped smoking included in this study is limited and may not be adequate to assess how smoke cessation would influence the immune response against *M*. *tuberculosis* (6 out of the 128 active TB patients who smoked stopped). However, results illustrated here strengthen the observation that quantity and length of tobacco smoking negatively impairs the immune system.

In conclusion, we here describe that (i) patients with active TB who smoke have a negative effect on radiological manifestations, sputum culture conversions in a dose-dependent manner, and treatment extension, (ii) tobacco smoke increases probability of false-negative IGRA results in active TB and LTBI patients due to decreased IFN-γ secretion, and (iii) IFN-γ response is affected by smoking being related with the pack-years consumption. Thus, this study adds further data about clinical performance according to tobacco smoke, which could help to explain and understand false-negative and indeterminate QFN-G-IT and T-SPOT.TB assays results on smokers. Furthermore, our data establish an association between tobacco and TB outcome due to a weaken host immune response caused by tobacco smoke. Advising smoke cessation and avoiding smoke exposure are two important measures for TB control [3, 42, 43]. Efforts to integrate smoking cessation interventions into TB directly DOT short-course have been performed improving the outcome of active TB patients [44, 45]. However, in spite of the efforts carried out in our study setting, only a low number of smoking patients with active TB quit tobacco smoking. Altogether making efforts on smoking cessation could improve quality life on TB patients and their clinical outcome of the disease.
